# Allogeneic amnion transplantation for the management of cutaneous graft-versus-host disease with associated ulcers: A promising therapeutic strategy

**DOI:** 10.1007/s00277-024-05990-8

**Published:** 2024-09-06

**Authors:** Andreas Siegmund, Daniel Wolff, Andrea Pagani, Marc Ruewe, Silvan Klein, Wolfgang Herr, Lukas Prantl, Sebastian Geis

**Affiliations:** 1 Department of Plastic, Hand, and Reconstructive Surgery, University Hospital Regensburg, Regensburg, Germany; 2 Department of Internal Medicine III, University Hospital Regensburg, Regensburg, Germany

**Keywords:** Graft-versus-host disease, Wound healing, Amniotic membrane, Leukemia, Blood disorders

## Abstract

Allogeneic hematopoietic stem cell transplantation (alloSCT) is the cornerstone treatment for various hematopoietic disorders, but its utility is often compromised by chronic graft-versus-host disease (cGvHD), affecting skin integrity and leading to ulcer formations. Traditional treatments, including systemic and topical therapies, frequently fail in severe cases. This study retrospectively examines three patients with therapy-resistant ulcers due to cGvHD post-alloSCT treated at the University Hospital of Regensburg in 2023. We evaluated the therapeutic impact of human amniotic membrane (hAM) transplantation—a novel approach utilizing hAM’s anti-inflammatory, anti-microbial, and anti-fibrotic properties for wound healing. Surgical debridement was followed by hAM application and routine follow-up. HAM transplantation led to complete wound closure in two out of three patients and a significant reduction in local pain and infection rates. The treatment alleviated the need for regular dressing changes within three months in two patients, demonstrating the hAM’s efficacy in fostering rapid and sustained healing. The utilization of hAM represents a promising alternative for the management of refractory skin ulcers in cGvHD patients, particularly when conventional methods are inadequate.

## Introduction

Allogeneic hematopoietic stem cell transplantation (alloSCT) stands as the primary curative intervention for various disorders of the hematopoietic system [1]. However, its effectiveness is countered by substantial morbidity and mortality, primarily attributed to the development of acute and chronic graft-versus-host disease (cGvHD) [2].

CGvHD manifests as a prolonged response of the donor’s immune system against the recipient’s tissues, affecting approximately 40% of patients following alloSCT [3]. The clinical presentations of cGvHD exhibit a diverse range of manifestations. This condition often affects the skin, sometimes resulting in the formation of difficult-to-treat ulcers in regions with extensive cutaneous sclerosis [4]. In severe cGvHD, mortality is frequently attributed to complications arising from infections [3]. The initial therapy consists of administering high doses of corticosteroids which are frequently combined with other immunosuppressive or immunomodulatory agents [5].

In the event of unsuccessful systemic and topical treatments, allogeneic skin transplantation from related donors in case of complete hematopoietic donor cell chimerism becomes a viable option [6]. Since the majority of patients stem cell donors are transplanted from unrelated donors and therefore are not eligible for donor skin graft donation, less invasive approaches are being sought.

This work presents the potential of human amniotic membrane (hAM) transplantation as a novel therapeutic approach for wound healing in patients [7] suffering from ulcers related to cGvHD. The hAM, derived from the placenta, possesses unique properties including anti-inflammatory, anti-microbial, and anti-fibrotic effects, which are conducive to wound repair and regeneration [7]. The utilization of hAM as a natural wound dressing has predominantly been confined to ophthalmology [7]. However, the recent introduction of hAM products has expanded its application to a wider range of indications, notably in the treatment of chronic non-healing wounds [8]. Notably, the utilization of hAM grafts in the treatment of granulating wounds and burns has been documented for over a century [8].

## Materials and methods

Three patients with cGvHD after alloSCT were evaluated retrospectively after receiving hAM (Deutsche Gesellschaft für Gewebetransplantation) for treatment of cGvHD related skin ulcers in 2023 at the University Hospital of Regensburg. Two patients had therapy-resistant ulcers due to severe cutaneous cGvHD, while one patient with cutaneous cGvHD experienced a deep defect following resection of a soft tissue tumor. Surgical debridement was employed to prepare the ulcers, followed by a single application of hAM onto the wounds. Subsequently, a sterile wound dressing was applied. The first wound check and bandage change took place three days after the surgery. Following that, routine wound inspections, dressing changes, and follow-up assessments were conducted during our consultation hours.

## Results

In 2 out of 3 patients, complete wound closure was achieved. All 3 patients experienced an improvement in local pain symptoms. Two out of 3 patients had no further wound infections. After 3 months, dressing changes were no longer necessary in 2 out of 3 patients.

### Patient 1

**Fig. 1:**
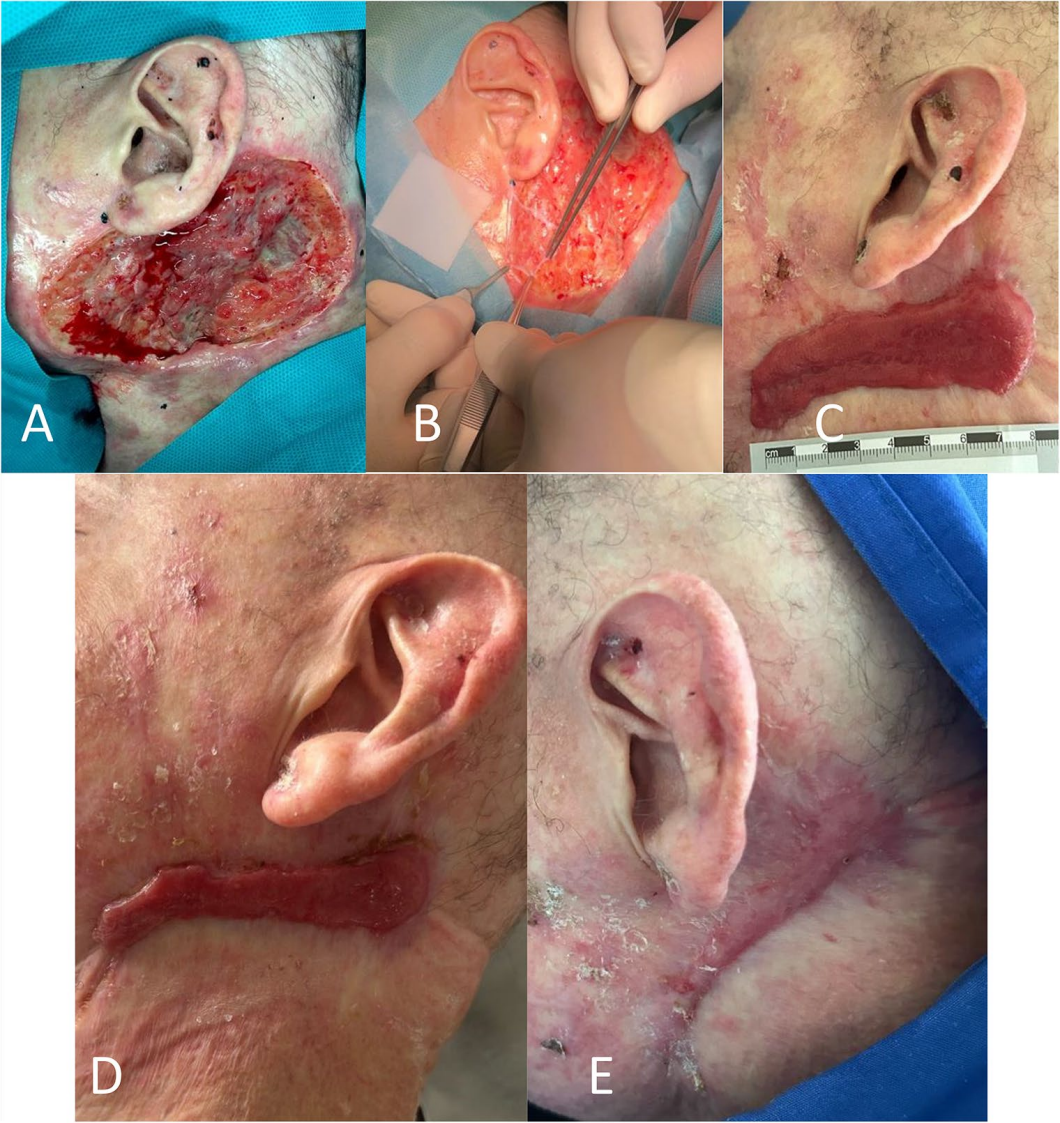
(**A**) Shows the deep wound with varying depth in the neck area after re-resection of the pleomorphic sarcoma. (**B**) Shows the transplantation of the hAM. **(C**) After 12 weeks, a hypertrophic scar and closed wound conditions are evident. (**D**) That scar continued to progressively decrease in size (4 months post-surgery). (**E**) Ultimately, 5 months after transplantation, scarred but completely closed wound conditions were observed

Patient 1 received in 2016 an alloSCT from an HLA-matched (10/10) unrelated donor for advanced chronic lymphatic leukemia. One year after transplantation, the patient developed severe cGvHD affecting the skin, while undergoing prednisolone and cyclosporine therapy. Despite treatment with ECP, prednisolone, MMF and cyclosporine, the GvHD continued to progress. Introduction of Ibrutinib in 2020 resulted in a reduction of inflammation and partial healing of the ulcers but repetitive severe bacterial infections. Therefore, cyclosporine and ibrutinib was terminated and tocilizumab started, which resulted in stabilization of cGvHD. In December 2022, a dermal mass on the left neck raised suspicion of spinocellular carcinoma, leading to surgical excision. However, histopathological analysis revealed the presence of a pleomorphic sarcoma. Re-resection resulted in a 12cmx5cm large defect which was covered by hAM. After transplantation, the patient experienced no wound pain (rated 0/10) and encountered no local infections. Over time, a hypertrophic scar developed and gradually diminished in size over time. Within a timeframe of 5 months, complete closure of the resection area was achieved (Fig. 1.).

### Patient 2

**Fig. 2:**
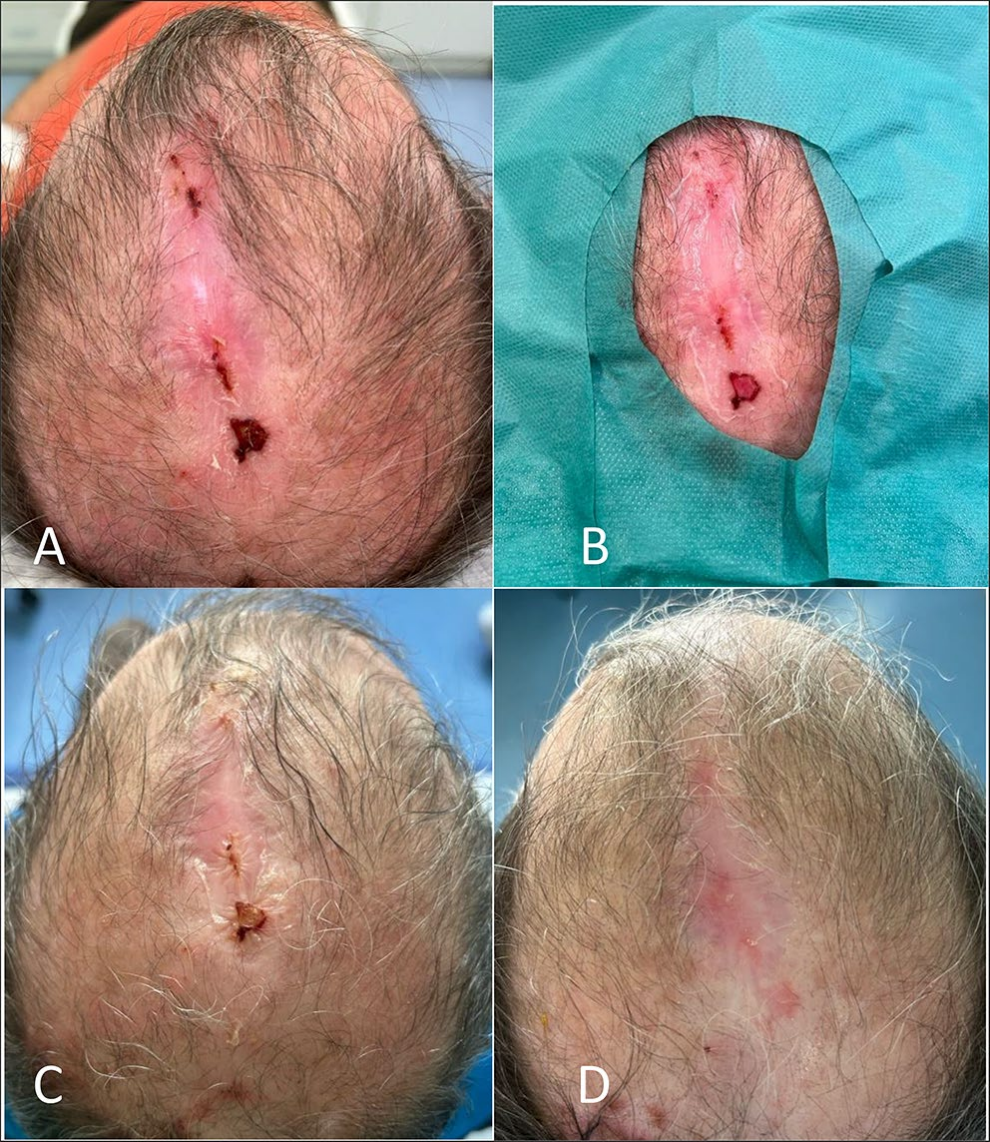
(**A**) shows the partially located wound on the head before transplantation, (**B**) the wound with the transplanted amniotic membrane. (**C**) After one week, the wound appears dry, with the amnion graft still visible. (**D**) After four weeks, a scarred healed, closed wound is visible

In 2004, the 55 year old male patient underwent an allogeneic SCT from an HLA-identical unrelated donor for AML in 1st complete remission. One year after transplantation he developed severe chronic GvHD affecting the skin, fascia and eyes. Treatment involved the use of steroids and ECP. In 2009 treatment for cGvHD was terminated. Through the next 10 years the patients developed refractory ulcers on the head which repetitively superinfected with Staph. aureus. Despite reinstitution of immunosuppression due to progressive cGvHD in 2021 with ECP, MMF and starting 2022 with Axatilimab, the painful ulcers remained unchanged. Therefore, after successful treatment of the infection, hAM transplantation was performed to address a parietal-occipital skin defect in 2023. Following transplantation, the patient reported complete resolution of pain and exhibited no signs of local infections throughout the procedure. Subsequently, the wound size diminished, and complete healing was achieved (Fig. 2.).

### Patient 3

**Fig. 3:**
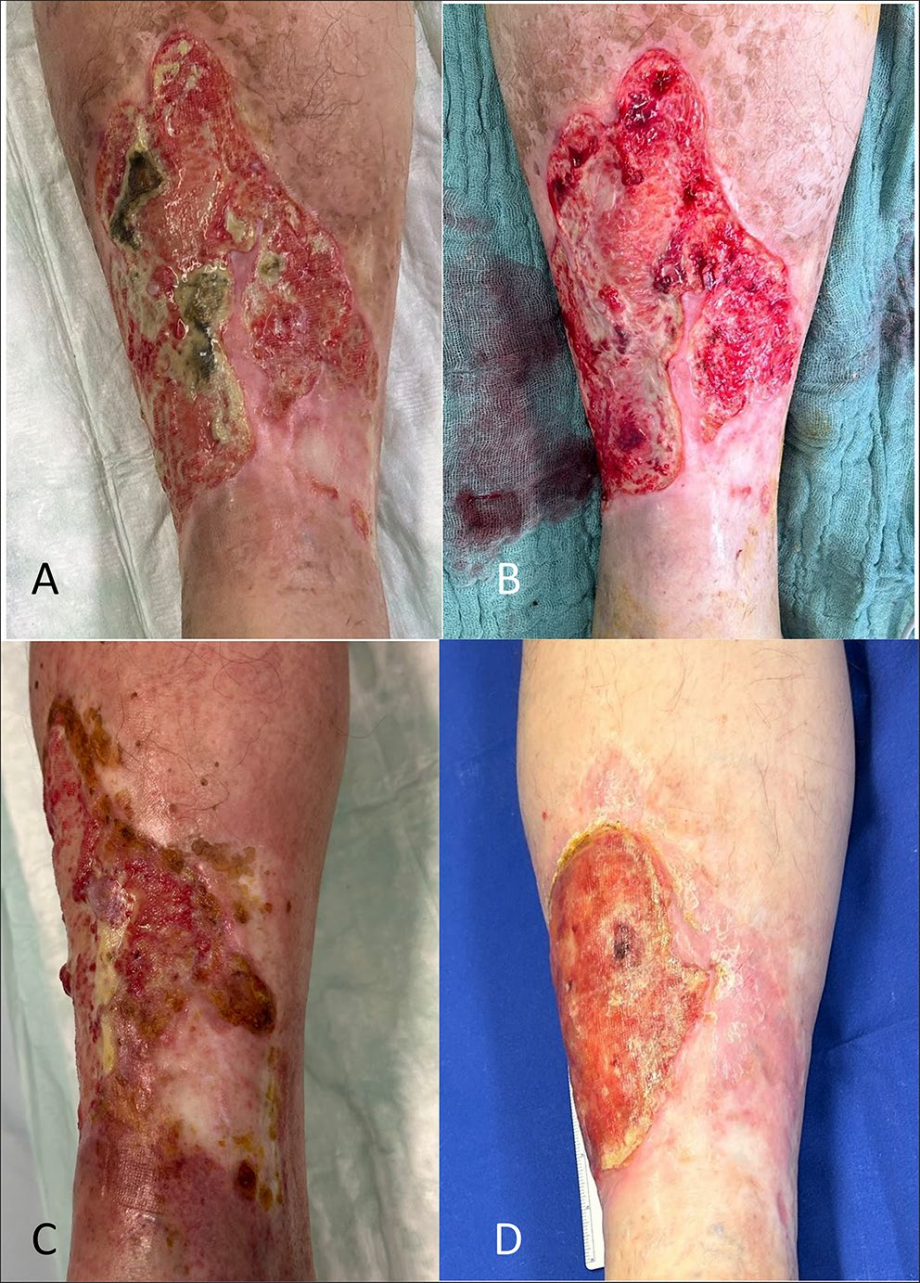
Illustrated are the stages of the wound on the right lower leg: (**A**) prior to surgical debridement, (**B**) post-debridement, (**C**) at 6 weeks following hAM transplantation, and (**D**) at 10 weeks post-transplantation. While complete wound closure was not attained, there was notable reduction in size, particularly in the medio-distal region of the wound

In 2019, the 60 year old male patient received an alloSCT after dose reduced conditioning for secondary AML following high dose chemotherapy and autologous HSCT for multiple myeloma from an HLA-identical brother. Subsequently, the patient developed acute GvHD grade 3 and during the further course severe steroid-refractory cGvHD with cutaneous deep sclerosis resulting in refractory ulceration on the right lower leg. The further course was complication with multiple episodes of bacterial superinfections. Systemic immunosuppressive treatment with steroids, ruxolitinib and ixazomib had no effect on cGcHD. Calcineurin-inhibitors and methotrexate were contraindicated due to renal impairment. In November 2023, hAM was applied to the ulceration on the patient’s right lower leg (Fig. 3.**).** Following the transplantation, there was a notable decrease in pain. Prior to the procedure, the patient reported a resting pain level of 6–7 out of 10 on a numeric rating scale, which decreased to 0 out of 10 post-hAM transplantation. However, the wound subsequently became reinfected with a 4MRGN pseudomonas 1 month after the transplantation. Despite this setback, there was a significant reduction in the wound area during the treatment process, accompanied by increased granulation tissue formation at the wound bed on new immunosuppressive treatment with belumosudil.

## Discussion

Ulcers in patients with refractory cutaneous cGvHD pose a therapeutic dilemma, stemming from persistent inflammation leading to prolonged tissue fibrosis, which includes vascular damage and subsequently hampers microcirculation [9]. Additionally, any inflammatory state carries the potential for the recruitment of alloreactive immune cells, perpetuating the existing inflammation and leading to compromised healing of skin ulcers [10]. The disease proves challenging to address using conventional therapeutic approaches. Patients experience pain, necessitating regular wound checks and dressing changes, along with managing associated complications. Local infections may arise, potentially leading to hospitalization if they pose systemic concerns.

J. Ammer (2012) and P. Lamby (2019) demonstrated successful treatment of refractory ulcers through the transplantation of split skin grafts allografting from related original stem cell donors [6, 11]. Unfortunately, this therapeutic option is not feasible in case of unrelated donors which represents the majority of patients affected. To enhance the healing of ulcers, mitigate chronic inflammation, and lower the risk of infection, the application of hAM onto chronic ulcers is a viable option. HAM supports epithelialization and showcases characteristics such as anti-fibrotic, anti-inflammatory, anti-angiogenic, and anti-microbial properties. Its non-immunogenic nature is a crucial aspect that enhances its suitability for grafting. Moreover, hAM has the capability to sustain a physiologically moist microenvironment, thereby minimizing humidity loss and fostering the process of wound healing [7, 12] which was clinically confirmed in our patients. Moreover, hAM has the potential to decrease local wound infections and thereby also reduced wound pain. Lastly, it’s noteworthy that 2 of the 3 patients achieved complete wound closure, eliminating the necessity for routine wound checks and dressing changes. In conclusion, hAM transplantation emerges as a potential option for cGvHD patients when conventional therapies prove ineffective.

## Data Availability

Derived data supporting the findings of this study are available from the corresponding author upon reasonable request.
